# Writing of strain-controlled multiferroic ribbons into MnWO_4_

**DOI:** 10.1038/s41467-021-26451-0

**Published:** 2021-10-27

**Authors:** Shingo Toyoda, Manfred Fiebig, Lea Forster, Taka-hisa Arima, Yoshinori Tokura, Naoki Ogawa

**Affiliations:** 1 RIKEN Center for Emergent Matter Science (CEMS), Saitama, 351-0198 Japan; 2 Department of Materials, ETH Zurich, 8093 Zurich, Switzerland; 3 Department of Advanced Materials Science, University of Tokyo, Kashiwa, 277-8561 Japan; 4 Tokyo College, University of Tokyo, Tokyo, 113-8656 Japan; 5 Department of Applied Physics, University of Tokyo, Tokyo, 113-8656 Japan; 6 PRESTO, Japan Science and Technology Agency (JST), 332-0012 Kawaguchi, Japan

**Keywords:** Ferroelectrics and multiferroics, Magnetic properties and materials

## Abstract

Local and low-dimensional structures, such as interfaces, domain walls and structural defects, may exhibit physical properties different from the bulk. Therein, a wide variety of local phases were discovered including conductive interfaces, sheet superconductivity, and magnetoelectric domain walls. The confinement of combined magnetic and electric orders to spatially selected regions may be particularly relevant for future technological applications because it may serve as basis of electrically controllable magnetic memory devices. However, direct observation of magnetoelectric low-dimensional structures cannot readily be done partly because of the lack of experimental techniques locally probing their physical nature. Here, we report an observation of multiferroic ribbon-like domains in a non-multiferroic environment in MnWO_4_. Using optical second harmonic generation imaging, we reveal that a multiferroic phase is stabilized by locally generated strain while the bulk magnetic structure is non-multiferroic. We further find that the confined multiferroic state retains domains with different directions of electric polarization and we demonstrate deterministic writing of a multiferroic state embedded in a non-multiferroic environment.

## Abstract (web summary)

It is difficult to find magnetoelectric low-dimensional structures partly due to the lack of experimental techniques locally probing their physical nature. Here, the authors observe multiferroic ribbon-like domains in a non-multiferroic environment in MnWO_4_ by optical second harmonic generation.

## Introduction

In geometrically frustrated magnets, magnetic order is suppressed due to competing magnetic interactions, and nontrivial magnetic structures such as spin-liquids^1^, skyrmions^2^, and spin-spirals^3^ may emerge. Among them, incommensurate non-collinear spin structures are of particular interest as they can show spin-induced electric polarization and pronounced magnetoelectric cross-coupling^4,5^. As mentioned above, local structures^6–10^ can exhibit new local phases^11,12^ so that interest has been shifting from bulk multiferroics towards multiferroicity confined to the lower dimensions with multiferroic domain walls as a prime example^13,14^.

We report an observation of deterministic multiferroic ribbons in a non-multiferroic environment in MnWO_4_ using optical second harmonic generation (SHG) as an imaging technique. Even though the bulk magnetic structure features a collinear non-multiferroic spin order, a multiferroic spin-spiral phase is locally stabilized due to the strain caused by surface defects in the crystal lattice. Within the multiferroic ribbons, we find polarization domain states in line with the ferroic nature of the spatially restricted phase. We propose a model explaining such a confined multiferroic state in a non-multiferroic environment, and we demonstrate multiferroic writing of information.

## Results

**Fig. 1:**
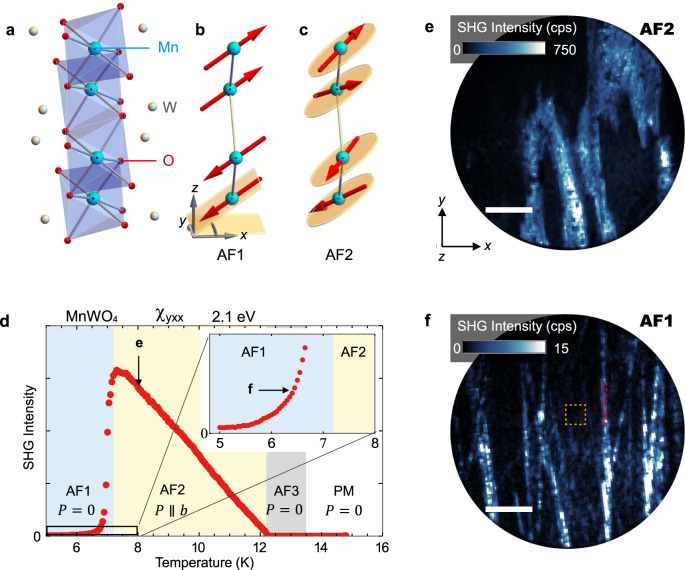
Temperature dependence of second harmonic generation. **a** Crystal Structure of MnWO_4_. **b**, **c** Magnetic structures in the **b** AF1 and **c** AF2 phases, respectively. The red arrows represent the magnetic moments of the Mn^2+^ ions and yellow circles show the spin-spiral plane. **d** Temperature dependence of the SHG for the tensor component ${\chi }_{{yxx}}$ at the photon energy $2\hslash \omega =2.1$ eV. The inset shows the enlarged view of the SHG intensity in the SHG-forbidden AF1 phase. **e** The SHG image was obtained at 8 K in the multiferroic AF2 phase after the zero-field cooling. **f** The SHG image was obtained at 6.5 K in the non-multiferroic AF1 phase. The orange and red regions indicate the regions which are used for the polarization dependence measurements in Fig. 2d. Scale bars, 200 μm.

MnWO_4_ crystallizes in the monoclinic wolframite structure with the centrosymmetric space-group *P2/c* (point group: 2/m). The lattice constants *a*, *b*, *c*, and $\beta$ are 4.82, 5.75, 4.99 Å and 91°, respectively^15^. In the following, we use Cartesian coordinates *x* $\parallel$ *a*, *y* $\parallel$ *b*, *z* $\parallel$ *c*, to represent the crystallographic axes, since $\beta \approx 90^\circ$. The magnetically active ion is Mn^2+^ (*S* = 5/2), which forms zigzag chains along the *z* axis, as shown in Fig. 1a. Several higher-order magnetic interactions are known to compete with one another, resulting in magnetic frustration^16^. Successive magnetic phase transitions below the Néel temperature (${T}_{N}=13.5$ K) reflect the presence of several near-degenerate antiferromagnetic phases. Coming from lower temperatures, these antiferromagnetic phases are called AF1 (collinear, commensurate, Fig. 1b), AF2 (elliptical spiral, incommensurate), and AF3 (collinear, incommensurate). Although the magnetic structures in the AF1 and AF3 phases retain the centrosymmetric point group 2/m symmetry, the spin-spiral structure in the AF2 phase lowers the magnetic point group to noncentrosymmetric 2. A magnetically induced ferroelectric polarization which is explained by the spin current model^17^ or an inverse Dzyaloshinskii-Moriya effect^18^ appears along the *y* axis in the multiferroic AF2 phase (Fig. 1c)^19,20^, whereas the AF1 and AF3 phases are not multiferroic.

Our experiment relies on spatially resolved imaging by SHG, that is, frequency-doubling of a light wave in a material. This is expressed by ${{{{{{\bf{S}}}}}}}_{{{{{{\rm{i}}}}}}}(2\omega )={\epsilon }_{0}{\chi }_{{ijk}}{{{{{{\bf{E}}}}}}}_{{{{{{\rm{j}}}}}}}(\omega ){{{{{{\bf{E}}}}}}}_{{{{{{\rm{k}}}}}}}(\omega )$, where ${{{{{\bf{S}}}}}}(2\omega )$, ${\chi }_{{ijk}}$, and ${{{{{\bf{E}}}}}}(\omega )$ are the source of the frequency-doubled light, the SHG susceptibility, and the electric field of the incident light, respectively. In the leading electric-dipole order, ${\chi }_{{ijk}}$ becomes nonzero in noncentrosymmetric materials only, which makes SHG a particularly powerful technique to detect the multiferroic state because of the associated breaking of spatial inversion symmetry. Therefore, only the AF2 phase permits an ED-SHG signal for which the tensor components ${\chi }_{{yxx}}$, ${\chi }_{{yyy}}$, and ${\chi }_{{xyx}}$ are nonzero with light propagating along the z axis^21,22^. Figure 1d shows the temperature dependence of the SHG signal of a (001)-oriented polished MnWO_4_ platelet at the photon energy $2\hslash \omega =2.1$ eV for ${\chi }_{{yxx}}$, which couples to the ferroelectric polarization along the *y* axis^23^. A large SHG signal whose amplitude scales with the spontaneous polarization is obtained in the multiferroic AF2 phase. An SHG signal, in total on the order of 1%, remains in the non-polar AF1 phase, see Fig. 1d. This residual signal cannot be associated with trivial sources such as surface SHG, luminescence, or higher-order non-polar SHG contributions, because it is absent in the paramagnetic and the AF3 phases. Therefore, the “forbidden” SHG signal likely is of magnetic origin and suggests the existence of a polar magnetic sub-structure in the non-multiferroic AF1 phase.

We perform SHG imaging measurements to understand the origin of the forbidden signal. Figure 1e, f show spatially resolved SHG images at 2.1 eV in the multiferroic AF2 phase and in the non-multiferroic AF1 phase, respectively. In the AF2 phase (Fig. 1e), a ferroelectric multidomain structure with bulk domains of a few hundred micrometers extension in all three dimensions is formed^24^. The different levels of brightness result from the interference of the SHG light waves from vertically arranged +*P* and –*P* domains. The SHG image in the AF1 phase (Fig. 1f) reveals that the forbidden SHG signal comes from line-shaped structures (“ribbons”). As mentioned, their polar nature must be related to magnetic order, because the associated SHG signal disappears in the paramagnetic phase. We thus reveal the existence of spatially confined polar magnetic regions in a non-polar magnetic bulk environment.

**Fig. 2:**
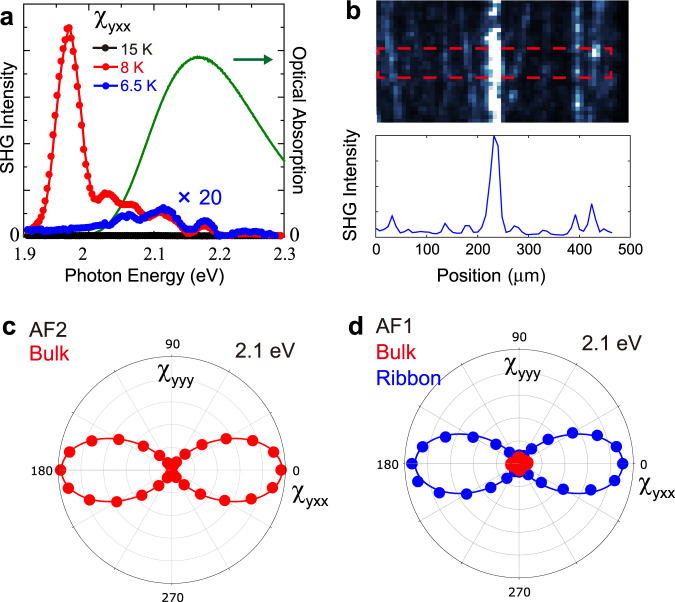
SHG spectrum, line profile, and polarization dependence. **a** SHG spectrum measured for the tensor component ${\chi }_{{yxx}}$. The green line shows the optical absorption spectrum measured at 15 K. **b** Line profile (red outline) of the SHG intensity at 6.5 K in the AF1 phase. **c** and **d** SHG polarization anisotropy with the polarization of ${E}^{2\omega }{||\; y}$ measured with varying polarization of fundamental light in the **c** multiferroic AF2 phase and **d** non-multiferroic AF1 phase. The SHG intensity from the AF1 dark bulk region (red) is one order of magnitude weaker than that in the AF1 multiferroic ribbons (blue). Solid lines are fits of the tensor component ${\chi }_{{yxx}}$.

To further elucidate the origin of the ribbons, we performed SHG spectroscopy and polarization anisotropy measurements. Figure 2a shows the SHG spectra for the tensor component ${\chi }_{{yxx}}$ at three different temperatures along with a linear optical absorption spectrum in the paramagnetic phase. The large SHG peak at 1.97 eV in the multiferroic AF2 phase is caused by optical phase matching^25^, whereas the structure at higher photon energy is assigned to intra-atomic spin-forbidden *d*-*d* electronic transitions of the Mn^2+^ ion^26^. Although the former signal stems from the entire thickness of the sample, the latter one is emitted only from a region near the surface because of the finite coherence length and the optical absorption band above 2 eV. Strikingly, the phase-matched SHG peak at 1.97 eV from the entire sample is reduced by three orders of magnitude in the AF1 phase, suggesting that only 0.1% of the crystal volume continues to emit AF2-like SHG and stemming from the near-surface region. In contrast, the signal at higher photon energy is reduced by only one order of magnitude. This suggests that the SHG signal in the AF1 phase stems from the near-surface region. This would locate the magnetic polar ribbons in the AF1 phase at this surface to which they appear to be pinned in some form. We show in Fig. 2b, a line profile of the SHG intensity in the AF1 phase. The SHG intensity in between ribbons is more than an order of magnitude weaker than that in the ribbons in agreement with the bulk-AF1-SHG-inactive collinear magnetic structure.

In the anisotropy plots in Fig. 2c and d, we show the polarization dependence of the SHG signal, which was measured by rotating the polarization of incident light while detecting frequency-doubled component polarized parallel to the *y* axis. Figure 2c shows the anisotropy in the AF2 phase, whereas Fig. 2d probe the AF1 phase. The SHG anisotropy in the bulk region (red dots) in Fig. 2d is obtained on the area of the dark bulk region (orange outline in Fig. 1f), whereas the SHG anisotropy of the ribbons (blue dots) is obtained on the area of the bright line-like region (red outline in Fig. 1f). In the multiferroic AF2 phase, the magnetically induced ferroelectric polarization along the *y* axis manifests as an SHG signal from the ${\chi }_{{yxx}}$ component, see Fig. 2c. Exactly the same type of SHG anisotropy was observed for the polar ribbons in the non-polar AF1 phase, which suggests an AF2-like confined phase with a spontaneous electric polarization along the *y*-axis as its origin. In contrast, the SHG signal in the dark AF1 bulk region is negligibly weak compared with that of the ribbons. This is in perfect agreement with the centrosymmetric magnetic structure of the AF1 phase where the leading-order electric-dipole SHG is forbidden^23^.

**Fig. 3:**
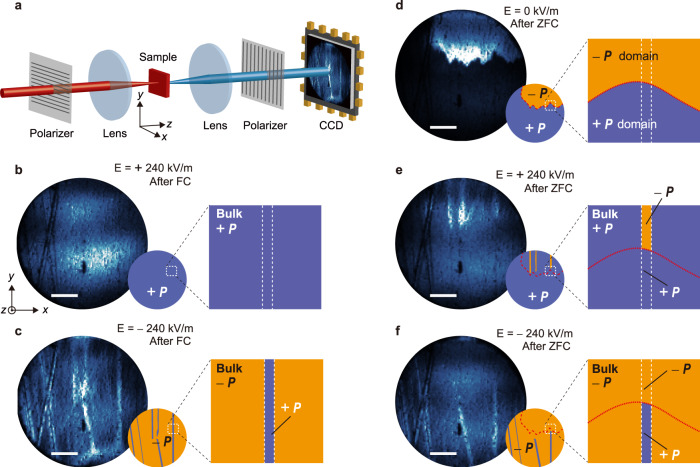
Multiferroic nature of the ribbons in the bulk multiferroic AF2 phase. **a** Schematic of the experimental setup for SHG imaging measurements. The incident light with the photon energy $\hslash \omega$ = 1.05 eV was polarized along the *x* axis. The SHG light with the polarization of ${E}^{2\omega }{||\; y}$ (tensor component ${\chi }_{{yxx}}$) was used for the imaging. The electric field was applied along the *y* axis, which is parallel to the ferroelectric polarization direction. **b**, **c** SHG images at 10 K (the bulk AF2 phase) obtained after field cooling at $+240$ kV/m in fields of **b** $+240$ kV/m and **c** $-240$ kV/m. **d**–**f** Electric field dependence of the SHG images at 10 K after zero-field cooling obtained in fields of **d** $0$ kV/m, **d** $+240$ kV/m, and **e** $-240$ kV/m. Scale bars are 200 μm.

The observed AF2-like nature of the ribbons in the AF1 phase indicates that a sort of phase pinning takes place at the surface. It is possible that the mechanism stabilizing this pinning in the AF1 phase is still active in the AF2 phase at higher temperatures. We, therefore, investigated the relationship between the AF2-like regions and the actual AF2 phase with the experimental setup shown in Fig. 3a. As in Fig. 1, we probe our sample in the quasi-phase-matched regime^27^. Figure 3b shows a spatially resolved SHG image obtained after cooling in an electric field $E=+240$ kV/m along the *y* axis. The homogeneous distribution of the SHG intensity indicates a single-domain state in which the ribbons have seemingly been lost. In a reversal of the electric field subsequent to the field cooling procedure, however, the polar ribbons reappear as bright stripes, see Fig. 3c. The opposite brightness indicates the electric polarization of the ribbon is now opposite from the polarization in the bulk. Hence, we find that the bulk polarization is flipped with the reversal of the electric field while the polarization of the ribbons remains parallel to the initial polarization direction $+{P}$. It demonstrates that the ribbons are robust against the external electric field, which supports the aforementioned conclusion of surface pinning.

Next, we investigate the electric field dependence of the SHG image after zero-field cooling (ZFC). As depicted in Fig. 3d, a multidomain state is formed in the multiferroic AF2 phase after ZFC, and the ribbons appear to have vanished. When we apply an electric field $E=+240$ kV/m, however, the upper part of the ribbons reappears whereas the lower part remains absent. Upon field reversal, the lower part of the ribbons becomes visible whereas the upper part vanishes. These results indicate that, most strikingly, the upper part of the ribbons has the $-{P}$ polarization, whereas the lower part has the $+{P}$ polarization after ZFC. Apparently, the ribbons can feature domains with opposite directions of the electric polarization. Since the existence of domains is a hallmark of ferroic order, their observation clearly shows that the polar ribbons in the AF1 phase can in fact be associated with a true ferroelectric and, hence, multiferroic phase. A possible mechanism that could stabilize such a state near the surface is strain. It was reported that the AF1–AF2 phase transition temperature is strongly affected by hydrostatic pressure, suggesting a large spin-lattice coupling in MnWO_4_^28^. Hence, surface strain, which may be present in the vicinity of crystallographic defects may suppress the phase transition from the AF2 phase to the AF1 phase, which would then stabilize a spatially confined multiferroic state in its bulk non-multiferroic environment. In the supplementary, we show that in the present sample grooves as a result of the sample polishing are the most likely source of such strain.

**Fig. 4:**
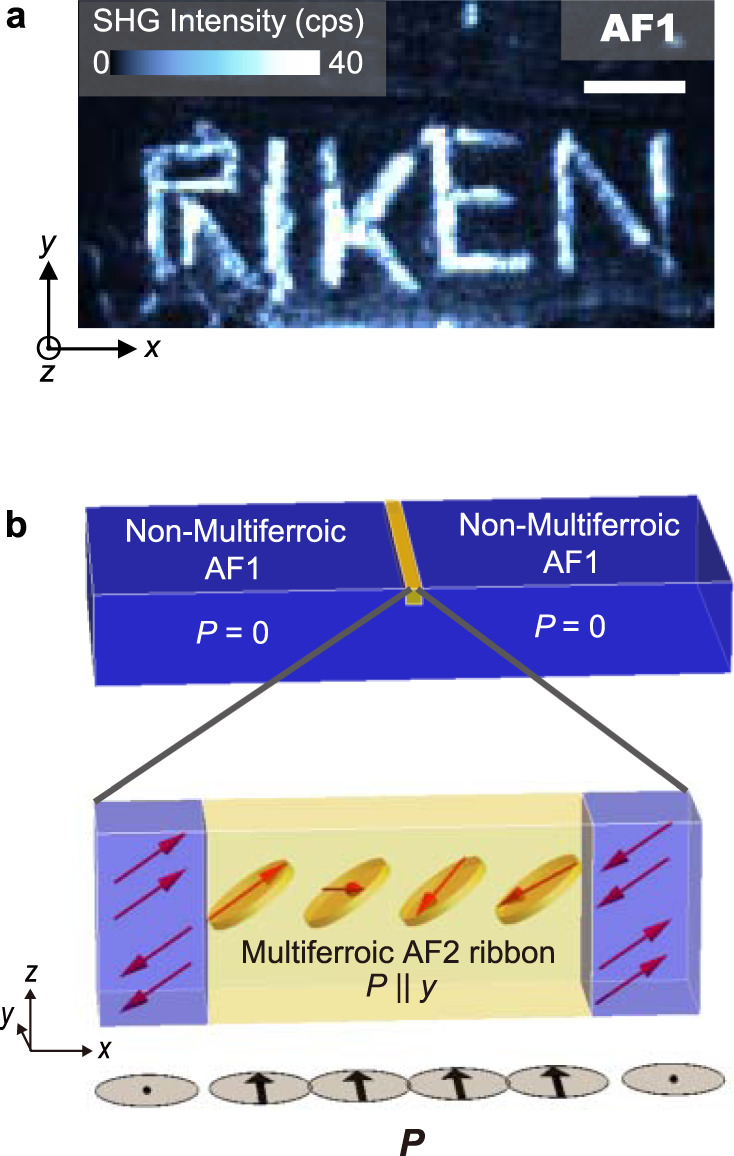
Controlled writing of the multiferroic state into a non-multiferroic environment. **a** SHG image at the photon energy $2\hslash \omega =2.1$ eV obtained at 6.5 K in the non-multiferroic AF1 phase. Letters were drawn onto the crystal surface with a scriber. Scale bar, 200 μm. **b** Model of the AF1/AF2 phase coexistence. The bulk magnetic structure is of the collinear non-multiferroic AF1 type, whereas the spatially confined ribbons show the AF2 multiferroic spiral spin structure. Note that the width of the AF1–AF2 phase boundary can be zero as the onset of the magnetic spiral and, hence, of the magnetically induced electric polarization occurs immediately after crossing from the non-multiferroic into the multiferroic phase.

In contrast to this accidental occurrence, the coupling between strain and multiferroicity allows us to create the multiferroic phase at specified locations with a scriber. In Fig. 4a, we show an SHG image in the non-multiferroic AF1 phase of a new (001)-oriented sample that clearly demonstrates our ability to write multiferroic structures in a highly controlled manner. We propose a model for the locally restricted AF2 phase in Fig. 4b. Interestingly, the phase boundary between the bulk-AF1 phase and the confined AF2 phase does not require a transitional region of finite width for this model magnetic structure. When entering the confined AF2 phase from the collinear AF1 phase, the spins immediately begin to rotate from unit cell to unit cell to reveal the multiferroic AF2 spin spiral. Note that in contrast to the AF1–AF2 boundary, the AF2–AF2 domain walls exhibit width on the order of 10 nm^29^.

To conclude, we have experimentally proven the existence of multiferroic regions in the form of ribbons within a non-multiferroic environment for which strain is likely responsible. We confirmed that these ribbons stabilize domains with opposite electric polarization which is robust against external electric fields. The proposed strain-multiferroic coupling enabled us to write spatially controlled multiferroic patterns into a non-multiferroic template, thus establishing a highly controlled scheme to seed magnetoelectric functionalities at the local level.

## Methods

### Sample preparation

Single crystals of MnWO_4_ were grown by the floating zone melting method. Stoichiometric amounts of powders of MnO (5.533 g) and WO_3_ (18.083 g) were mixed. A polycrystalline rod of MnWO_4_ was synthesized by a solid-state reaction by heating at 1000 °C for 18 hours in the air. The single-crystal growth was carried out in airflow with a feed speed of 3 mm/h by using an infrared radiation furnace. The obtained crystal was oriented using Laue X-ray diffraction patterns and cut into thin plates with the widest faces (001). Sample thickness is 200 μm for the measurements in Figs. 1–3, whereas it is 1.7 mm for the SHG image in Fig. 4a. The crystal defects on the sample surface were naturally applied during the polishing procedure for the former sample, whereas we intentionally applied them for the latter sample by using a scriber at room temperature. The scriber was installed on an XYZ stage with a precise position adjustment, which allows for writing the letters at specific positions on the sample surface.

### SHG imaging

The light source used for our SHG imaging measurements is a regeneratively amplified laser, producing 190 fs light pulses at 6 kHz repetition rate. The wavelength of the laser was tuned with an optical parametric amplifier. The sample was mounted on a copper holder with a hole diameter 1 mm in a liquid-helium-cooled variable-temperature cryostat. The SHG data points and images were taken in a transmission geometry using a 200 mm telephoto lens for projection and a liquid-nitrogen-cooled CCD camera as a detector.

### Reporting summary

Further information on research design is available in the Nature Research Reporting Summary linked to this article.

## Supplementary information

Supplementary Information

Lasing Reporting Summary

## Data Availability

The data that support the findings of this study are available from the corresponding authors on reasonable request.
