# Supplementary material for: Writing of strain-controlled multiferroic ribbons into MnWO4
Source: Nat Commun. 2021 Oct 27;12:6199. doi: 10.1038/s41467-021-26451-0 (PMC8551292; doi:10.1038/s41467-021-26451-0)
Supplement: Supplementary file 1 — Supplementary Information [file 41467_2021_26451_MOESM1_ESM.pdf]

Supplementary Information

Writing of strain-controlled multiferroic ribbons into $MnWO_4$

Shingo Toyoda[1*], Manfred Fiebig[1,2*], Lea Forster[2], Taka-hisa Arima[1,3], Yoshinori Tokura[1,4,5], and Naoki Ogawa[1,5,6]

[1] RIKEN Center for Emergent Matter Science (CEMS), Saitama 351-0198, Japan

[2] Department of Materials, ETH Zurich, 8093 Zurich, Switzerland

[3] Department of Advanced Materials Science, University of Tokyo, Kashiwa 277-8561, Japan

[4]Tokyo College, University of Tokyo, Tokyo 113-8656, Japan

[5]Department of Applied Physics, University of Tokyo, Tokyo 113-8656, Japan

[6]PRESTO, Japan Science and Technology Agency (JST), 332-0012, Kawaguchi, Japan

*E-mail: shingo.toyoda@riken.jp, manfred.fiebig@mat.ethz.ch

Supplementary Note 1. Comparison between the SHG image and the polarization-microscope image

Supplementary Figures 1a and b show the comparison between the polarization-microscope image at room temperature and the SHG image obtained at 6.5 K in the non-multiferroic AF1 phase, respectively. We observed several linear defects applied to the surface in the polarization-microscope image, and most of the SHG in the AF1 phase is emitted in the vicinity of these defects. This supports the surface pinning scenario described in the main text, where the spatially confined multiferroic domains are stabilized due to the strain around these defects. However, the SHG intensity is not proportional to the brightness of the linear defects in the polarization-microscope image. For example, some of the clearly visible linear defects in the polarization-microscope image are barely visible in SHG image. This implies that the brightness in the polarization-microscope image does not generally correspond to the magnitude of the strain. Other contributing effects can be optical diffraction on the scratches, the geometry of the scratches (deep vs. wide), the way in which the strain was applied (blunt instrument vs. scriber), etc.

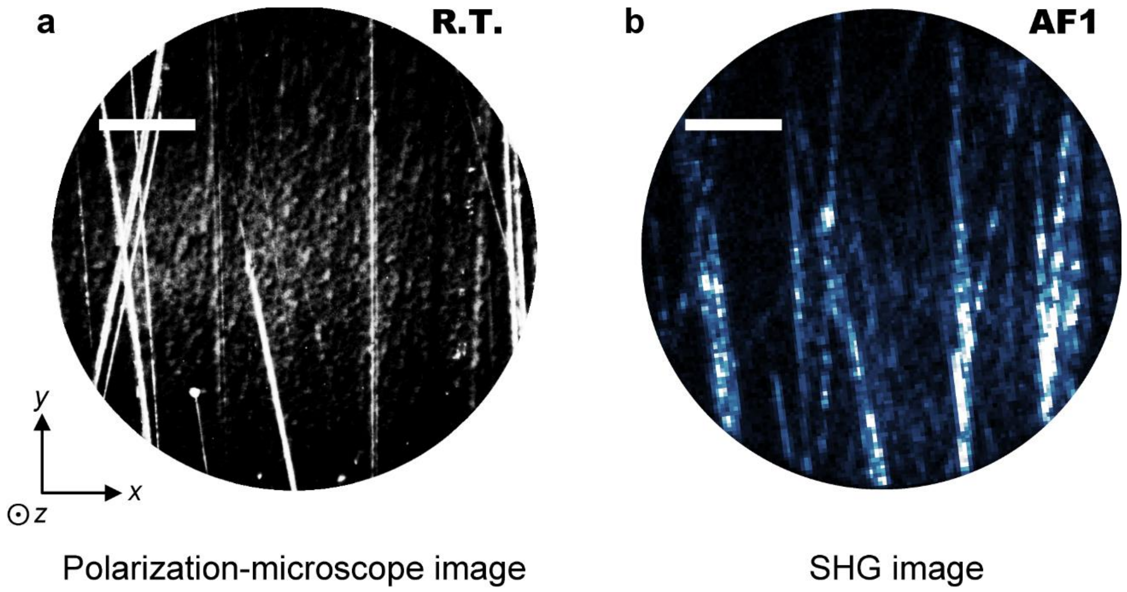

Supplementary Figure 1. Correlation between the surface defects and multiferroic ribbon structures. **a**, Polarization-microscope image obtained with a white-light source in the same region as the SHG image in (b). For setting the polarization of the light, we chose a crossed-nicols configuration to highlight the linear defects applied to the sample surface. **b**, The SHG image was obtained at 6.5 K in the non-multiferroic AF1 bulk phase. Scale bars are 200 μm.

Supplementary Note 2. SHG temperature dependence in the sample with applied surface damage

To demonstrate that the multiferroic ribbons in the non-multiferroic environment are promoted by the strain around linear surface defects, we intentionally applied additional defects to the surface by sand paper and measured the effect on the SHG intensity. Supplementary Figure 2a shows temperature dependence of the SHG intensity before and after applying the defects. The temperature evolution of the SHG intensity in the multiferroic AF2 phase shows the same line shape and, in particular, slope, confirming that our treatment did not affect the overall bulk properties. This allowed us to use the temperature where the onset of the SHG signal occurs as calibration point for correcting the small set-up-related temperature shift between the two measurements in Supplementary Fig. 2a. However, we observed a slight shift of the AF1-AF2 transition temperature towards lower temperature and an increase of the remaining SHG signal within the AF1 bulk phase. To further investigate the origin of the shift of the transition temperature, we measured the spatial distribution of the SHG signal remaining in the AF1 bulk phase. The SHG image in Supplementary Fig. 2b shows that the emitted SHG signal stems from all over the sample surface. This is in sharp contrast to the behavior of the original sample in Supplementary Fig. 1b, where only several straight lines in the vicinity of the surface defects emit SHG light. The increase of the SHG-active regions indicates that the surface is now covered with a dense distribution of the applied defects, such that all the remaining undamaged area of the surface is now strained and thus pushed into the AF2 phase.

Therefore, the multiferroic local domains are now covering the entire surface and contribute to SHG, leading to the pseudo-shift of the AF2/AF1 transition temperature.

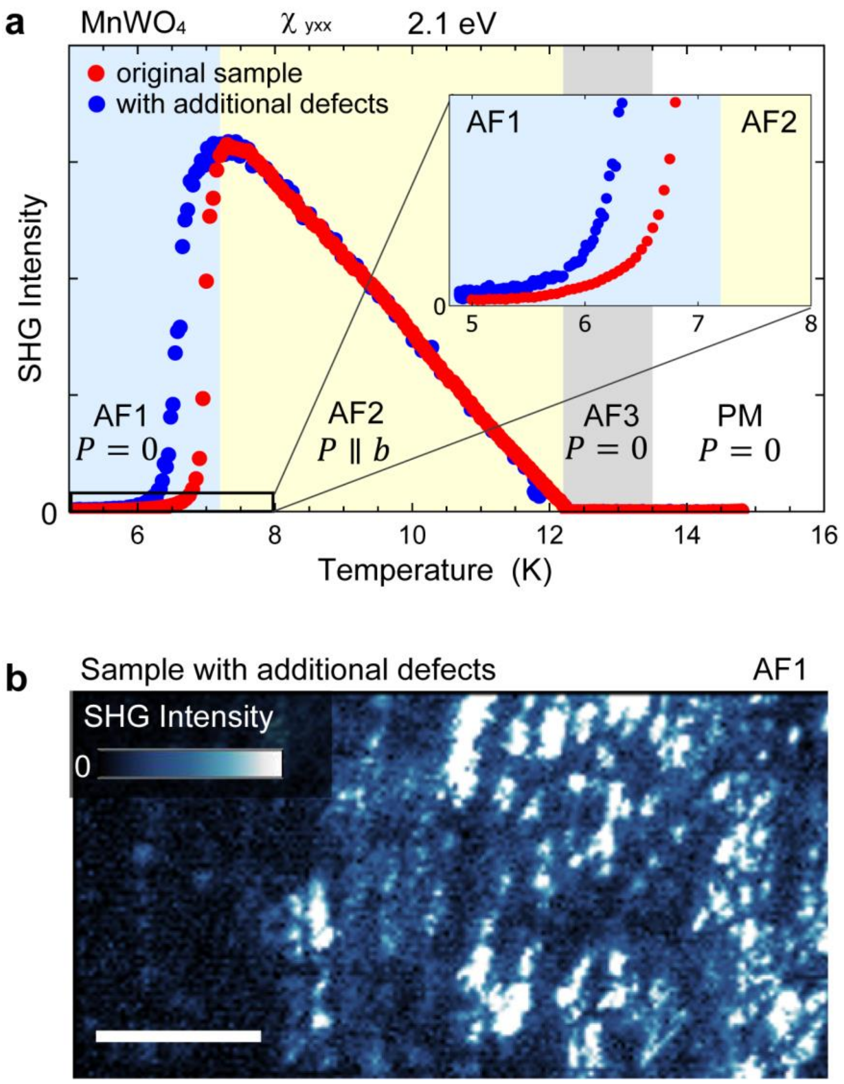

Supplementary Figure 2. The effect of the surface defects on the SHG temperature dependence. a, Temperature dependence of the SHG intensity for the tensor component χ_{yxx} at the photon energy $2\hbar\omega = 2.1$ eV for the same sample before (red) and after (blue) adding the surface defects. Both data sets are normalized using the maximum SHG intensity. The small temperature difference between the two measurements was corrected as described in the section S2. The inset

shows the enlarged view of the SHG intensity in the AF1 phase. **b**, The SHG image for the sample with the additional surface defects in the non-multiferroic AF1 phase. Scale bar is 200 μm.
